# TransfersomILs: A Synergy to Boost the Skin Delivery of Hydroxycinnamic Acids

**DOI:** 10.3390/pharmaceutics18080962

**Published:** 2026-08-05

**Authors:** Ana Júlio, Marta B. Martins, Teresa Martinho, João Vieira, Nuno Saraiva, Catarina Rosado, Catarina Pereira-Leite

**Affiliations:** 1 CBIOS, ECTS, Universidade Lusófona, Campo Grande, 376, 1749-024 Lisboa, Portugal; ana.julio@ulusofona.pt (A.J.); p8490@ulusofona.pt (M.B.M.); p6769@ulusofona.pt (J.V.); nuno.saraiva@ulusofona.pt (N.S.); 2 ECTS, Universidade Lusófona, Campo Grande, 376, 1749-024 Lisboa, Portugal; 3 Universidad de Alcalá, Departamento de Ciências Biomédicas, Ctra. Madrid-Barcelona km. 33,600, 28805 Alcalá de Henares, Madrid, Spain; 4 Laboratório Associado para a Química Verde (LAQV), REQUIMTE (Rede de Química e Tecnologia), Faculty of Pharmacy, University of Porto, Rua de Jorge Viterbo Ferreira n. 228, 4050-313 Porto, Portugal

**Keywords:** hydroxycinnamic acids, hybrid nanosystems, transfersomes, ionic liquids, skin application, transfersomILs

## Abstract

**Background/Objectives**: Innovative topical delivery systems are needed to improve the stability, loading capacity, and performance of poorly water-soluble bioactive compounds. TransfersomILs, hybrid nanosystems combining transfersomes with ionic liquids (ILs), represent a promising strategy for this purpose. This work assessed the effect of incorporating cholinium-based ILs into transfersomal formulations loaded with hydroxycinnamic acids (HCAs)—ferulic, caffeic, and *p*-coumaric acids. **Methods**: TransfersomILs were prepared by the thin-film hydration method followed by sonication, with or without HCA incorporation. Formulations were characterised in terms of physicochemical properties, storage stability and impact on keratinocyte viability. In vitro release, permeation, and occlusion studies were also performed. **Results**: IL incorporation significantly improved formulation performance. TransfersomILs showed smaller vesicle sizes and more negative zeta potential values than conventional transfersomes, indicating improved physicochemical characteristics. ILs also increased association efficiency and loading capacity for all HCAs, although the magnitude depended on both the IL and the compound. Release profiles were compound-dependent, reflecting distinct release kinetics due to variable HCA–IL–membrane interactions. Permeation studies showed enhanced HCA flux across both silastic and human epidermal membranes compared with aqueous solutions and/or conventional transfersomes, with [Cho][Gly] generally showing superior performance. All formulations demonstrated acceptable cytocompatibility and occlusive properties. **Conclusions**: The combination of transfersomes and cholinium-based ILs demonstrated a synergistic effect, highlighting transfersomILs as a versatile platform for improving the topical delivery of HCAs.

## 1. Introduction

The use of nanosystems has been proposed as an advantageous strategy for addressing skin permeation, given the need for efficient delivery systems that enable active pharmaceutical ingredients to overcome this challenging biological barrier [1,2]. They not only allow for a longer period of permanence on the skin but also better control of drug release, while ensuring improved delivery into the stratum corneum and protection of the loaded compound from chemical and/or physical deterioration [3].

Lipid-based nanovesicles have been widely explored for this purpose, providing promising clinical results and being considered ideal carriers for several compounds, due to their high biocompatibility and biodegradability [4,5]. Nevertheless, the disadvantages of traditional liposomes, such as limited skin permeation and storage stability [6], prompted the development of advanced nanovesicles for cutaneous applications, such as transfersomes.

Transfersomes differ from typical liposomes in that they contain a phospholipid component and a single-chain surfactant that functions as an edge activator (EA) [7]. EAs, when combined in the appropriate ratio with a suitable lipid, act as membrane-destabilizing agents, increasing the deformability of vesicle membranes, allowing transfersomes to become deformable and much more flexible, eventually resulting in higher skin permeation capability [8,9]. Thus, these vesicular systems overcome the primary downsides of conventional liposomes, since they can transport hydrophilic and/or hydrophobic molecules through pores that are considerably smaller than their own diameters into deeper epidermal layers, without compromising their own structural integrity [10,11,12]. However, transfersomes still have some drawbacks, such as poor storage stability and a tendency to aggregate over time [11], which may be circumvented by the inclusion of multifunctional excipients, namely ionic liquids (ILs).

ILs, usually found in a liquid state below 100 °C, are salts made up of both anions and cations, contrasting with inorganic salts by having a lower melting point due to a bigger size of either the anion, cation or both [13,14]. Through mechanisms such as lipid fluidization and extraction in the stratum corneum, ILs have been shown to enhance transcutaneous transport and can be considered a promising alternative to traditional organic solvents and surfactants in topical drug delivery systems [13,15]. They are also increasingly used as solvents/cosolvents, adjuvants or surfactants in the biopharmaceutical industry [16]. More specifically, in the pharmaceutical field, 3rd-generation ILs, which are developed from natural sources such as amino acids, have shown promising properties, particularly due to their favourable safety profile, high biocompatibility, and considerable solubilising capacity [17]. Among these, choline-amino acid ILs ([Cho][AA]) have emerged as multifunctional ingredients in skin formulations. Members of this IL family have been incorporated into emulsions [18], hydrogels [19,20], and nanodelivery systems [21], as recently reviewed by Pereira et al. [22]. (2-Hydroxyethyl)trimethylammonium phenylalaninate ([Cho][Phe]) and (2-hydroxyethyl)trimethylammonium glycinate ([Cho][Gly]) are representative examples of [Cho][AA] and were selected for the present study [18].

**Figure 1:**
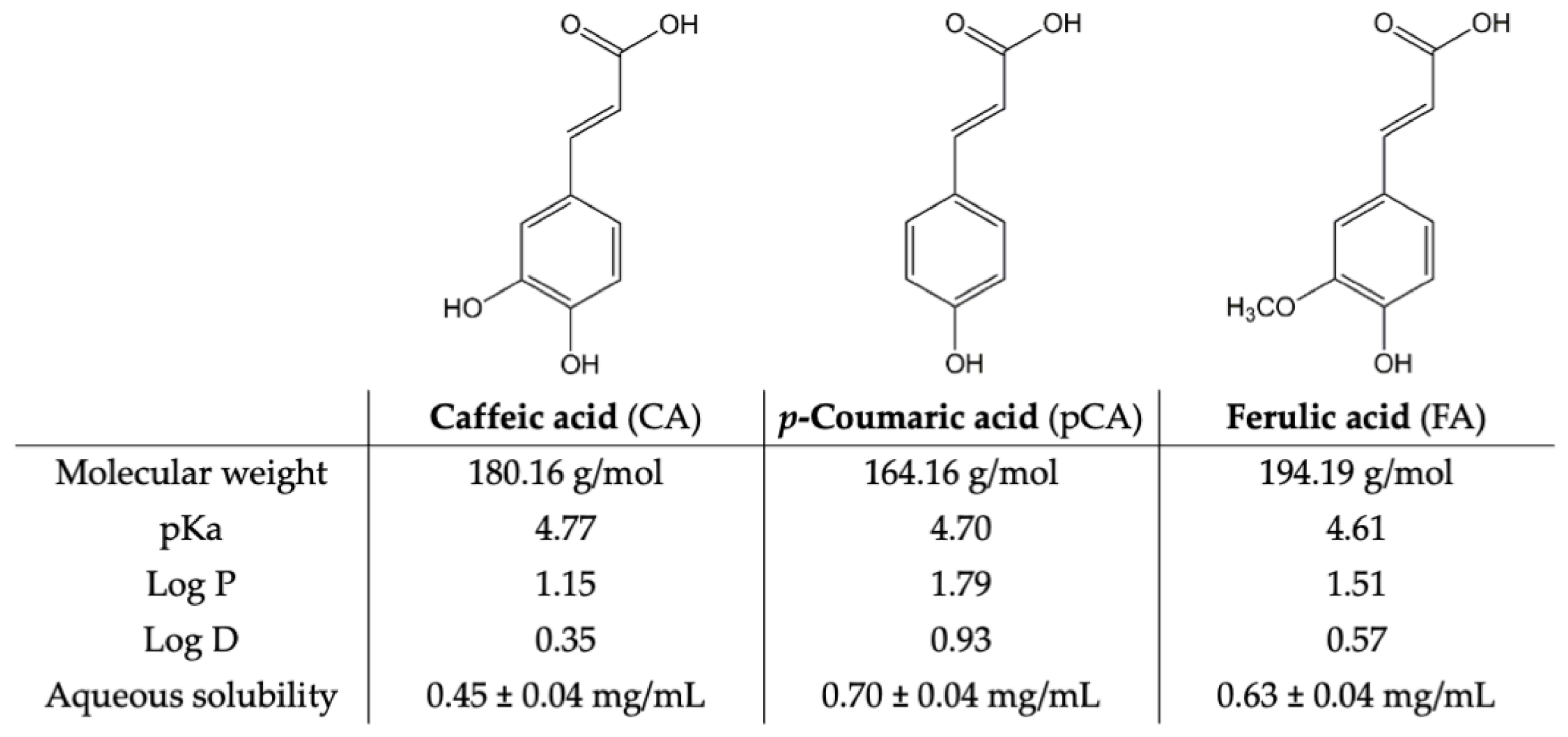
Chemical structure of the caffeic, *p*-coumaric and ferulic acids (CA, pCA and FA, respectively) and their most relevant physicochemical properties: molecular weight [30], dissociation constant (pKa) [31], lipophilicity expressed as log P [32] and log D (pH 5.5) [31] and aqueous solubility at 25 °C and pH 5.5 [18].

Nanosystems have been proposed as a strategy to promote the dissolution and encapsulation of antioxidant molecules, protecting them from degradation and ensuring a more sustained activity [23]. They can be particularly suitable for polyphenols, which are typically poorly stable and water insoluble [24]. Hydroxycinnamic acids (HCAs) are a group of natural phenolic acids widely distributed in plants and therefore found in vegetables, fruits, and tea [25,26,27]. This class of phytochemicals includes caffeic, *p*-coumaric, and ferulic acids (Figure 1) [25,28]. Despite their structural similarity, their physicochemical properties and biological activity are highly dependent on the position of the hydroxyl functional groups bound to the aromatic ring [29].

Caffeic acid (CA) is the major hydroxycinnamic acid found in the human diet [33]. CA has shown antimicrobial activity against bacteria, fungi and viruses [33]. Moreover, when applied in skin formulations, it promotes collagen production while also conferring antioxidant and anti-inflammatory activities [34]. *p*-Coumaric acid (pCA) is a phenolic acid with moderate lipophilicity, slightly higher than that of the other HCAs under study, and can be found in either free form or conjugated to other molecules [31]. Its biological activity spans antioxidant, antimicrobial, skin regeneration and anti-inflammatory activities [35]. Due to its free radical scavenging potential, pCA is also used in whitening formulations, providing an anti-melanogenic effect [35]. Ferulic acid (FA) displays strong anti-inflammatory, anti-microbial, and antioxidant activities [18,36]. Despite this, the use of this active compound is hindered by several challenges, including its rapid photodegradation [18].

Previous studies have shown that combining [Cho][AA] with HCAs improves their aqueous solubility and enhances their permeation across artificial membranes [18,37]. The synthesis of cholinium-based ILs from HCAs has also been proposed as an alternative strategy to improve the solubility of these phytochemicals [38,39]. However, to date, no studies have confirmed whether combining [Cho][AA] with HCAs improves their permeation through human skin.

Building on a previous proof-of-concept study on the development of transfersomILs [21], the present work further assesses the impact of incorporating cholinium-based ILs to enhance the performance of transfersomes loading HCAs. TransfersomILs incorporating [Cho][Phe] or [Cho][Gly] were designed to load three model HCAs with distinct biopharmaceutical properties (CA, pCA and FA), and were characterised in terms of physicochemical properties and storage stability. Envisioning topical cutaneous applications, the performance of the transfersomILs was assessed in vitro in terms of release, transcutaneous permeation, occlusion and cytocompatibility. In order to establish the impact of the ILs in these nanosystems, conventional transfersomes were used as benchmarks in all assays. To our knowledge, this is the first study to evaluate the impact of combining [Cho][AA] with transfersomes on key performance parameters of topical formulations loading HCAs, including skin permeation, occlusive properties, and keratinocyte compatibility.

## 2. Materials and Methods

### 2.1. Materials and Reagents

Tween^®^ 80, caffeic acid, *p*-coumaric acid, ferulic acid, and chloroform from Sigma–Aldrich (Saint Louis, MO, USA), soya lecithin (Alfa Aesar, Kandel, Germany), and methanol (Honeywell, Sulze, Germany) were used to produce transfersomes. Two cholinium-based ILs were produced in-house—(2-hydroxyethyl)trimethylammonium phenylalaninate ([Cho][Phe]) and (2-hydroxyethyl)trimethylammonium glycinate ([Cho][Gly]), as described elsewhere [18].

For the cell viability studies, trypsin, penicillin–streptomycin solution, dimethyl sulfoxide (DMSO), and thiazolyl blue tetrazolium bromide (MTT) were acquired from Sigma–Aldrich (Saint Louis, MO, USA) and Dulbecco’s modified Eagle’s medium (DMEM) with high glucose and Foetal Bovine Serum (FBS) were purchased from Biowest (Nuaillé, France).

### 2.2. Production of TransfersomILs

The transfersomILs were produced using the lipid film hydration method with subsequent sonication. Each HCA was dissolved in chloroform:methanol (3:1, *v*/*v*) with soya lecithin (40 mg/mL) and Tween^®^ 80 (2.1 mg/mL, as EA). ILs were also dissolved in the same organic mixture. Using a rotary evaporator (Heidolph VV/WV 2000, Heidolph Instruments GmbH & Co., Schwabach, Germany) at 40 °C and 100 rpm for 10 min, the lipidic film was produced, followed by vacuum for 2 h to remove any traces of solvents. Afterwards, bidistilled water (pH 5.5) was used to hydrate the film, which was then vortexed and sonicated at 70% amplitude for 20 min using a ^1^/_4_″ (6.4 mm) probe and a Q125 Sonicator (QSonica Sonicators, Newtown, CT, USA) at room temperature. The nanosystems were finally equilibrated under shaking, using a VIBRAX-VXR (IKA, Jankel & Kunkel, Satufen, Germany), for 30 min at 200 rpm. Control transfersomes were prepared using the same protocol, without IL incorporation. The quantitative composition of the prepared formulations is described in Table 1. Three independent batches of each formulation were prepared for subsequent characterisation.

### 2.3. Characterisation of TransfersomILs

#### 2.3.1. Size, Polydispersity Index and Zeta Potential

Using the dynamic light scattering method with the Delsa^TM^ Nano C equipment (Beckman Coulter, Inc., Brea, CA, USA), the size and polydispersity index (PDI) of the nanosystems were characterised, after diluting the sample (50×) with bidistilled water. Three independent batches of each formulation were characterised over 70 measurement cycles, with each measurement performed in triplicate at room temperature (23 ± 2 °C).

The Zeta potential (ZP) was also analysed over 30 cycles at 23 ± 2 °C, with measurements performed in triplicate for each independent batch after diluting the sample (25×) in bidistilled water using Phase Analysis Light Scattering (PALS) on a Nanobrook Omni instrument (Brookhaven Instruments, Holtsville, NY, USA).

#### 2.3.2. Association Efficiency and Loading Capacity

An aliquot of the formulations was used to calculate both association efficiency (AE) and loading capacity (LC) by centrifuging it in Vivaspin^®^ 500 devices (Sartorius, Goettingen, Germany) with a 50 kDa filter for 40 min at 12,000× *g*, to separate the vesicles from the supernatant, following previously reported methodologies [40,41,42]. Then, the supernatant was used to quantify the non-loaded fraction of HCA by calibration curve analysis using a UV–Visible spectrophotometer (Evolution^®^ 300, Thermo Scientific, Hertfordshire, England) at 23 ± 2 °C. Then, AE and LC were determined by Equations (1) and (2). AE and LC were determined in duplicate for each of the three independent batches (n = 3).

(1) $$\%\mathrm{AE}=\frac{\left[Total\;HCA\right]-[Non-loaded\;HCA]}{\left[Total\;HCA\right]}\times 100$$

(2) $$\%\mathrm{LC}=\frac{\left[Total\;HCA\right]-[Non-loaded\;HCA]}{\left[Total\;Lipid\right]}\times 100$$

#### 2.3.3. Stability Studies

All formulations were stored in refrigerated conditions (5 ± 3 °C) for 60 days. After 15, 30, 45, and 60 days of storage, analyses of Size, PDI, and ZP were performed, as described in Section 2.3.1.

### 2.4. In Vitro Release Studies

A dialysis bag diffusion method was used to conduct in vitro release studies. Each formulation (1.5 mL) was transferred to a Spectra/Por^®^ 1 dialysis bag (molecular weight 6–8 kDa; Repligen^®^, Waltham, MA, USA), in duplicate. Each bag was then placed in phosphate-buffered saline (PBS) at pH 7.4 and stirred at 37 ± 2 °C to mimic physiological conditions. At several time points (0.5, 1, 2, 4, 6, 8, 10, 12, and 15 h), an aliquot of the external medium was taken and immediately replaced with the same volume of pre-heated PBS, while maintaining sink conditions. UV–Visible spectrophotometry (using an Evolution^®^ 300 spectrophotometer, Thermo Scientific, Hertfordshire, England) was used to evaluate the amount of HCA that was released at each time point, considering each compound’s maximum absorption wavelength. The cumulative amount of each HCA released, expressed as a percentage over time, was calculated to plot the release profiles. Drug release kinetics were evaluated using the zero-order, first-order, Higuchi, Korsmeyer–Peppas, and Hixson–Crowell models. These models describe concentration-independent release, concentration-dependent release, diffusion-controlled transport, diffusion/relaxation mechanisms, and release associated with changes in the surface area and geometry of the dosage form, respectively [43].

### 2.5. Permeation Assay

Permeation studies were conducted in two stages: first in silastic membranes (Liveo™ 7-4107, DuPont^TM^, Braine-l’Alleud, Belgium) and afterwards using human epidermis [44]. Human breast skin tissue was obtained after cosmetic reduction surgery, following informed consent and approval by the ethics committee of the Clinica Milénio (Lisbon, Portugal). Epidermal membranes were separated from full-thickness skin by thermal treatment at 60 °C and mounted on filter paper [45]. Membranes were placed between the donor and receptor compartments in Franz cells with a receptor volume of ≈4 mL, creating a diffusional area of 0.95 cm^2^. A mixture of PBS pH 7.4 and ethanol (75:25) was used to fill the receptor compartments, which were kept at a temperature of 37 ± 2 °C and under continuous stirring at 300 rpm, using a magnetic bar, while 500 μL of nanoformulation or saturated solution of HCA in water was applied in the donor compartment. Each formulation was tested with five replicates, comparing free and encapsulated HCA. Aliquots of the receptor phase (300 μL) were taken at predetermined time points: 3, 6, 9, 12, and 24 h. To determine the amount of permeated compound, each collected sample was placed in a 96-well plate, and the HCA absorption was measured at each compound’s maximum absorption wavelength using the microplate reader BioTek Synergy HTX (Agilent BioTek Instruments, Santa Clara, CA, USA). The flux values for each permeation experiment were determined by tracking the cumulative amount of HCA permeated and calculating the slope of the curve after achieving steady-state conditions [46].

### 2.6. Occlusion Studies

Following a prior protocol [40,47], the occlusion capacity of the formulations was assessed in vitro by weighing flasks filled with 5 mL of bidistilled water, registering the initial weight (initial weight, *w_i_*), and covering them with a polydimethylsiloxane (PDMS) membrane (Liveo™ 7-4107, DuPont^TM^, Braine-l’Alleud, Belgium). Then, 50 μL of either the formulation or bidistilled water (used as a control) was transferred to the top of each membrane, in quintuplicate. After this, the flasks were kept in an oven (U30 Memmert^®^, Lilienthal, Germany) set to 32 ± 2 °C for 24 h. Finally, the membranes were removed, and the flasks’ final weight was determined (*w_f_*). The percentage of water loss (%WL) of each flask was obtained considering the following equation:

(3) $$\%\mathrm{WL}=\frac{w_{i}-w_{f}}{w_{i}}\times 100$$

### 2.7. Cell Viability Studies

Cell viability studies were conducted using a human keratinocyte cell line (HaCaT) obtained from Cell Lines Service (Eppelheim, Germany). HaCaT cells were kept at 37 °C in a humidified air environment with 5% CO_2_, in culture medium (DMEM with high glucose level supplemented with 10% FBS and 1% penicillin–streptomycin). A volume of 200 μL of culture medium was used to seed 6 × 10^3^ cells per well in 96-well plates. After 24 h, cells were exposed to 10 μL of the formulations under investigation, which included loaded and unloaded formulations, for another 24 h. The positive (100% DMSO) and negative (bidistilled water) controls were tested under the same conditions as the studied formulations. Cell viability was assessed using the MTT reduction assay as previously described [48,49]. The absorbance values for the negative control cells were considered to correspond to 100% of cell viability. Two independent experiments, each using five replicate cultures, were performed. The sample size was defined by an a priori power calculation (α = 0.05; power = 90%) to distinguish cell viability values above or below the predefined 70% threshold following ISO 10993-5:2009 [50].

### 2.8. Statistical Analysis

The results were displayed as mean ± standard deviation (SD). Normality and homogeneity of variance were assessed prior to statistical analysis. Physicochemical properties at time 0 were compared using Student’s *t*-test, while stability data were analysed by repeated-measures ANOVA with Geisser–Greenhouse correction followed by Dunnett’s multiple comparisons test. Permeation and cell viability data were analysed by one-way ANOVA (with Welch’s and Brown–Forsythe corrections when appropriate) followed by Dunnett’s multiple comparisons test. Occlusion data were analysed using the Kruskal–Wallis test followed by Dunn’s multiple comparisons test. Statistical significance was set at * *p* < 0.05, ** *p* < 0.01, *** *p* < 0.001, and **** *p* < 0.0001. The analysis was carried out using GraphPad Prism version 10, an application created by GraphPad Software (Boston, MA, USA).

## 3. Results

To evaluate the impact of incorporating cholinium-based ILs into HCA-loaded transfersomILs, nanoformulations were prepared with and without each HCA and/or IL, namely [Cho][Phe] and [Cho][Gly]. For transfersomILs developed with CA or pCA, each HCA was loaded at the maximum solubility in water or in the corresponding water:IL mixture [18]. In a subsequent study, to determine whether the observed effects were due to the higher compound concentration enabled by the presence of the IL or to the incorporation of the IL itself, all FA-loaded transfersomILs were prepared using the maximum solubility of FA in water [18].

### 3.1. Characterisation of TransfersomILs

The produced transfersomILs presented average vesicle sizes ranging from 69 nm to 136 nm, while PDI was established between 0.20 and 0.25 and ZP between −20 mV and −48 mV (Table 2).

Regarding the impact on size (Table 2), by comparing the control blank transfersomes (T_B) with those loading an HCA (T_CA, T_pCA or T_FA), no significant influence was noted in the size of the nanoparticles encapsulating CA or pCA, but this parameter was slightly increased for FA. However, when the control transfersomes (T_B) were set against the blank transfersomILs (T_ChPhe or T_ChGly), a significant decrease in size was observed. The same trend was observed when loaded transfersomes (T_CA, T_pCA or T_FA) were compared with loaded transfersomILs, both for [Cho][Phe] (T_CA_ChPhe, T_pCA_ChPhe or T_FA_ChPhe) and [Cho][Gly] (T_CA_ChGly, T_pCA_ChGly or T_FA_ChGly).

Regarding PDI (Table 2), all transfersomILs presented an even distribution with values below 0.25, with no statistically relevant differences. In terms of ZP (Table 2), similarly to what happened with size, there were no relevant differences when comparing the unloaded and loaded transfersomes, but this parameter significantly decreased both for loaded and blank transfersomILs.

When comparing AE, the transfersomILs also performed better, but the increase was more pronounced with [Cho][Gly] for all HCAs, whereas concerning LC, it was more accentuated with [Cho][Phe] across all HCAs.

Phase-contrast microscopy images confirmed that both blank transfersomes and transfersomILs exhibited the expected spherical morphology (Figure S1).

### 3.2. Preliminary Stability Studies

**Figure 2:**
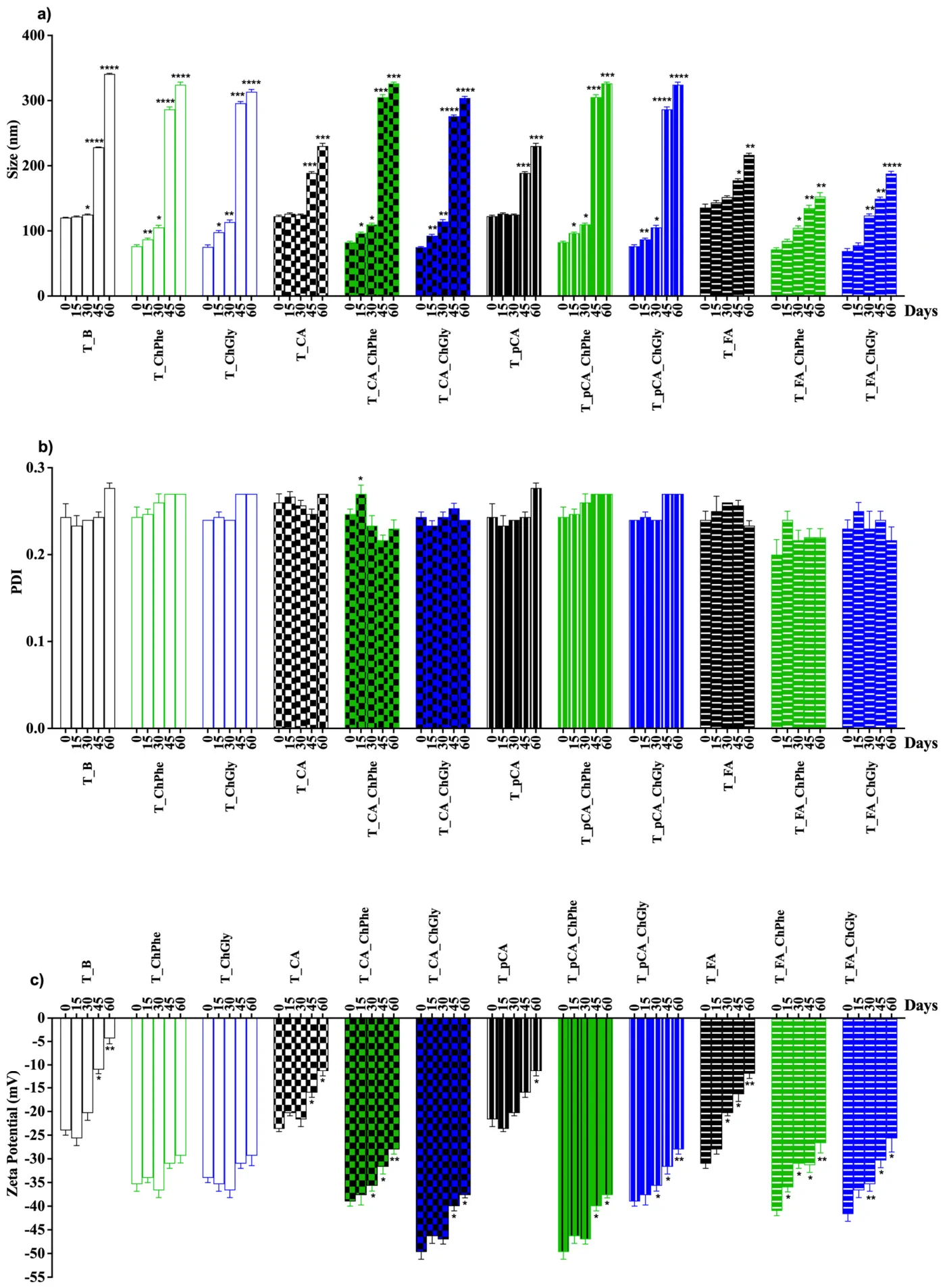
Variation in the (**a**) size, (**b**) polydispersity index (PDI), and (**c**) zeta potential of HCA-loaded and unloaded transfersomes in the absence or in the presence of each IL (n = 3, mean ± SD; * *p* < 0.05, ** *p* < 0.01, *** *p* < 0.001, and **** *p* < 0.0001 compared with day 0). T_B corresponds to blank transfersomes; T_CA, to caffeic acid (CA)-loaded transfersomes; T_pCA, to *p*-coumaric acid (pCA)-loaded transfersomes; T_FA, to ferulic acid (FA)-loaded transfersomes; T_ChPhe, to transfersomes containing [Cho][Phe], and T_ChGly, to transfersomes containing [Cho][Gly]. Transfersomes containing an HCA and an IL are identified by combining the corresponding abbreviations (e.g., T_CA_ChPhe).

The storage stability of the developed formulations was evaluated over 60 days by monitoring particle size, PDI, and ZP (Figure 2). Particle size increased over time for all formulations (Figure 2a), although the extent of this increase varied considerably depending on formulation composition. TransfersomILs based on FA maintained comparatively lower size increases, suggesting improved physical stability. In contrast, lower size increases were observed for CA and pCA when no IL was incorporated. Regarding PDI (Figure 2b), all formulations maintained relatively low values throughout storage, generally below 0.3, indicating a reasonably narrow particle size distribution and acceptable homogeneity. Only minor fluctuations were observed over the 60-day period, with no marked signs of severe polydispersity development. The evolution of ZP (Figure 2c) showed that all formulations maintained negative surface charges throughout the storage period. However, ZP values became progressively less negative over time, indicating a reduction in the surface charge of all transfersomal formulations. The incorporation of ILs contributed to more negative ZP values (<−30 mV) during storage, suggesting improved electrostatic stabilization. Nevertheless, this surface charge was insufficient to completely prevent particle aggregation, as evidenced by the increase in particle size observed over time.

### 3.3. In Vitro Release Studies

**Figure 3:**
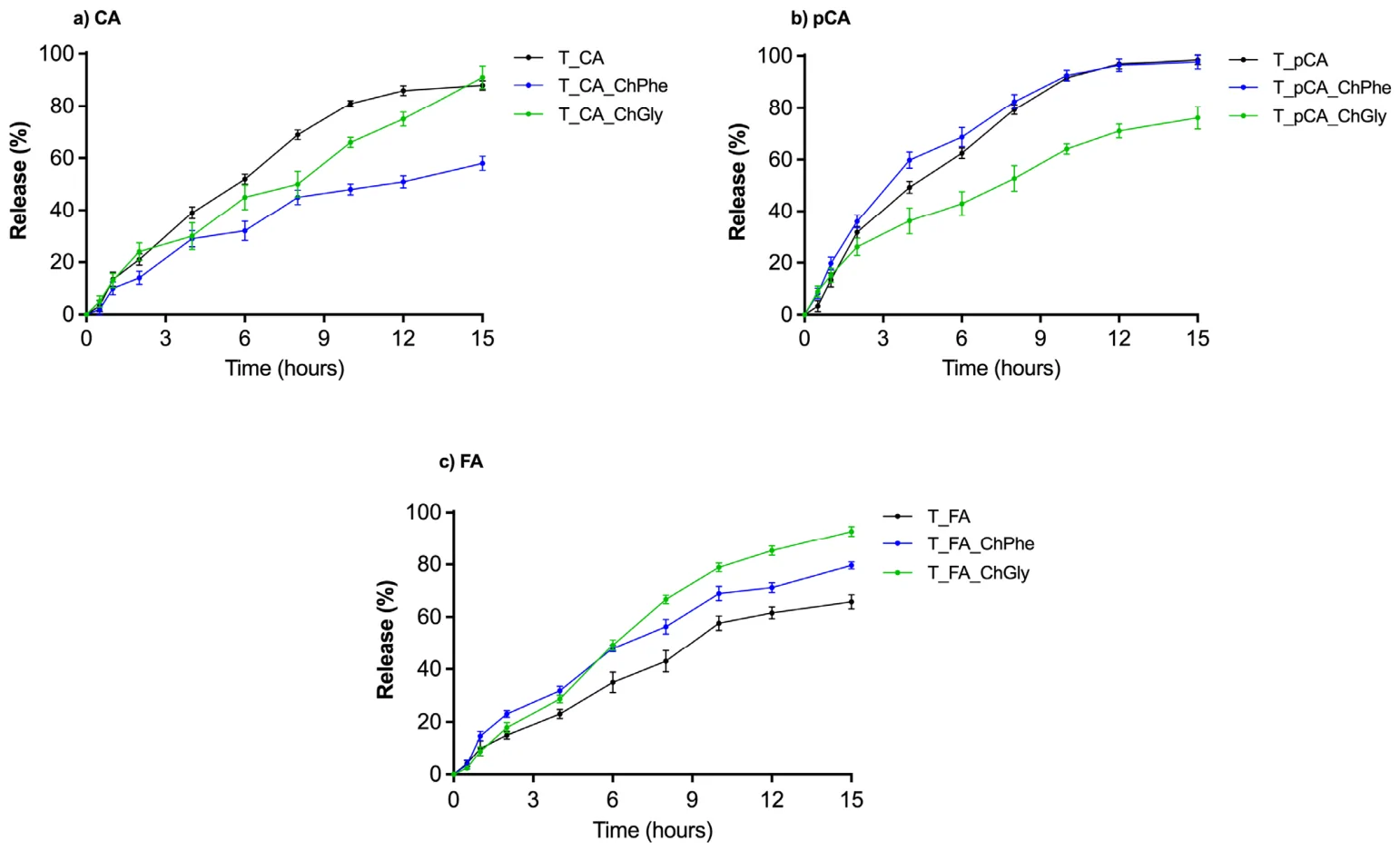
Release profile of (**a**) Caffeic acid (CA), (**b**) *p*-Coumaric acid (pCA), and (**c**) Ferulic acid (FA) from transfersomes in the presence of each IL, [Cho][Phe] (blue line) or [Cho][Gly] (green line), and in the absence of ILs (black line), during 15 h in a phosphate-buffered saline at pH 7.4 (n = 3, mean ± SD). Formulation designations combine the abbreviations of transfersomes (T), the HCA, and, where applicable, the IL (ChPhe for [Cho][Phe] and ChGly for [Cho][Gly]) (e.g., T_CA_ChPhe).

The release profile of the different compounds under study was traced for 15 h (Figure 3). Depending on the compound, the release profile appearance changed. Only the transfersomes loaded with CA and pCA and the [Cho][Phe] transfersomIL with pCA reached a plateau. The ILs seem to be affecting each HCA release profile differently. For instance, [Cho][Phe] delayed CA release but had little to no effect on pCA release and even promoted FA release.

The release profiles of CA, pCA and FA from transfersomes with and without ILs were fitted to the zero-order, first-order, Higuchi, Korsmeyer–Peppas, and Hixson–Crowell kinetic models (Table S1). For T_CA and T_FA, the highest correlation coefficients were obtained with the first-order model (R^2^ = 0.9824 and 0.9849, respectively), whereas T_pCA was best fitted by the Higuchi model (R^2^ = 0.9748), although the first-order (R^2^ = 0.9646) also showed good agreement. In contrast, the Hixson–Crowell model consistently yielded the lowest correlation coefficients for all three formulations (R^2^ = 0.6890–0.7380). The incorporation of ILs modified the release constants and, in some cases, altered the best-fitting kinetic model. For CA, both the first-order and Higuchi models remained the best fit after the incorporation of [Cho][Phe] (R^2^ = 0.9760 and 0.9778, respectively), with lower release constants than T_CA, whereas [Cho][Gly] shifted the release profile towards zero-order kinetics (R^2^ = 0.9853). For pCA, the incorporation of both [Cho][Phe] and [Cho][Gly] maintained the good fit of the first-order (R^2^ = 0.9839 and 0.9914, respectively) and Higuchi (R^2^ = 0.9763 and 0.9908, respectively) models, although the release constants decreased, particularly in the presence of [Cho][Gly]. For FA, the first-order model continued to provide the best fit (R^2^ = 0.9933 and 0.9777 for [Cho][Phe] and [Cho][Gly], respectively), and the incorporation of ILs increased the release constant, with the greatest increase observed for [Cho][Gly].

### 3.4. Permeation Assays

**Figure 4:**
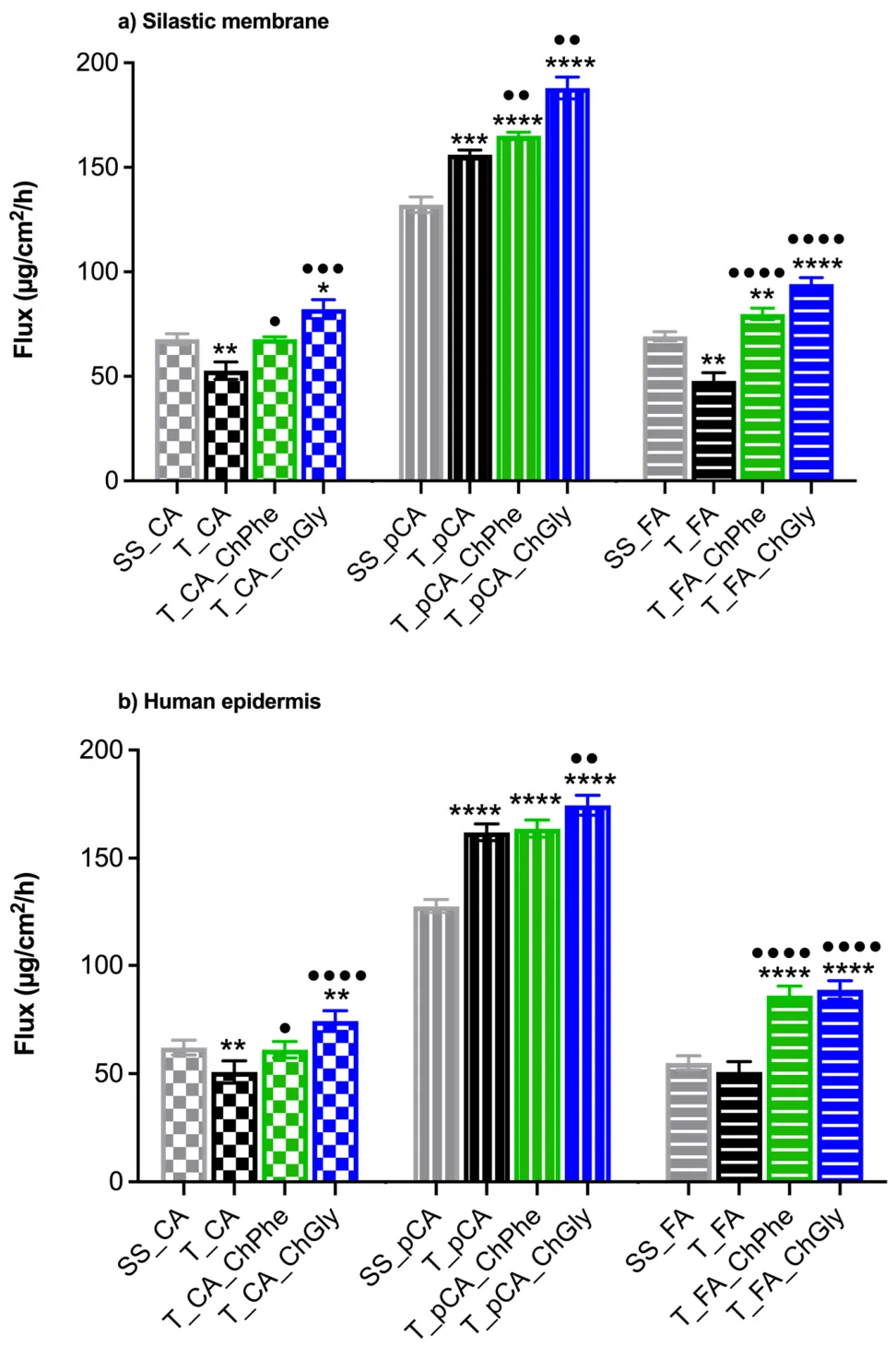
Permeation flux of each HCA in (**a**) silastic membrane or (**b**) human epidermis from an aqueous saturated solution or transfersomes in the absence or presence of each IL, [Cho][Phe] or [Cho][Gly], after 24 h using PBS pH 7.4:ethanol (75:25) as receptor medium. Donor-applied formulations are identified by combining the abbreviation for saturated solution (SS) or transfersomes (T) with the corresponding HCA (caffeic acid, CA; *p*-coumaric acid, pCA; ferulic acid, FA) and, where applicable, the IL (ChPhe for [Cho][Phe] and ChGly for [Cho][Gly]) (e.g., T_CA_ChPhe). Values are presented as mean ± SD, * *p* < 0.05, ** *p* < 0.01, *** *p* < 0.001, and **** *p* < 0.0001 compared with the respective saturated solution and • *p* < 0.05, •• *p* < 0.01, ••• *p* < 0.001, and •••• *p* < 0.0001 compared with the respective transfersome without IL.

Permeation studies were conducted comparing the performance of HCA-loaded transfersomes and transfersomILs against a saturated solution of each HCA in water. In the initial studies, a simplistic model of the stratum corneum was used—silastic membranes (Figure 4a)—whereas in the second set of permeation assays, human epidermis was employed (Figure 4b).

In the results obtained for transfersomILs, HCAs exhibited enhanced flux through the silastic membrane, thereby displaying higher permeation than transfersomes. [Cho][Gly] seemed to have a more relevant permeation enhancement effect, which was observed across all HCAs. In epidermal membranes, transfersomILs enabled higher fluxes compared with the transfersomes or saturated solution, corroborating the results obtained with silastic, but both ILs enhanced the permeation of HCAs, except in the case of the T_CA_ChPhe system when benchmarked with aqueous saturated solution. Interestingly, in both silastic and epidermis, the performance of transfersomes was inferior to that of aqueous solutions of CA and FA.

### 3.5. Occlusion Studies

**Figure 5:**
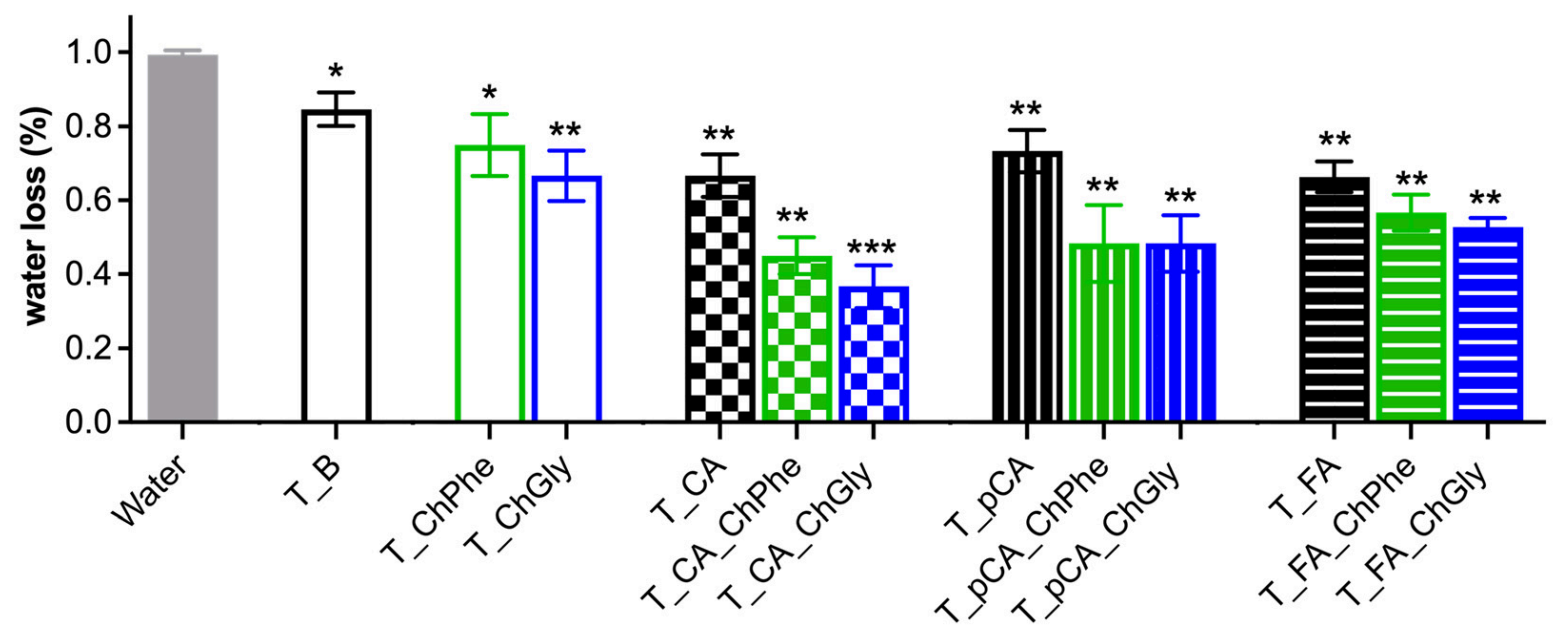
Occlusion effect, measured as water loss percentage, of transfersomes with and without each HCA (Caffeic acid, CA; *p*-Coumaric acid, pCA; and Ferulic acid, FA) in the presence or absence of [Cho][Phe] or [Cho][Gly] (n = 5, mean ± SD, * *p* < 0.05, ** *p* < 0.01, and *** *p* < 0.001 compared with water, used as control). Formulation designations combine the abbreviation for transfersomes (T) with, where applicable, the corresponding HCA and/or IL (ChPhe for [Cho][Phe] and ChGly for [Cho][Gly]) (e.g., T_CA_ChPhe). T_B denotes blank transfersomes.

The results obtained in this assay demonstrated that transfersomes alone are capable of creating an occlusion effect, which might be due to their lipidic components (Figure 5). Nevertheless, when comparing this with the results obtained with the unloaded transfersomILs, the occlusive effect is slightly more pronounced. Interestingly, in the case of loaded transfersomILs, statistically significant differences were noted across all HCAs.

### 3.6. Cell Viability

**Figure 6:**
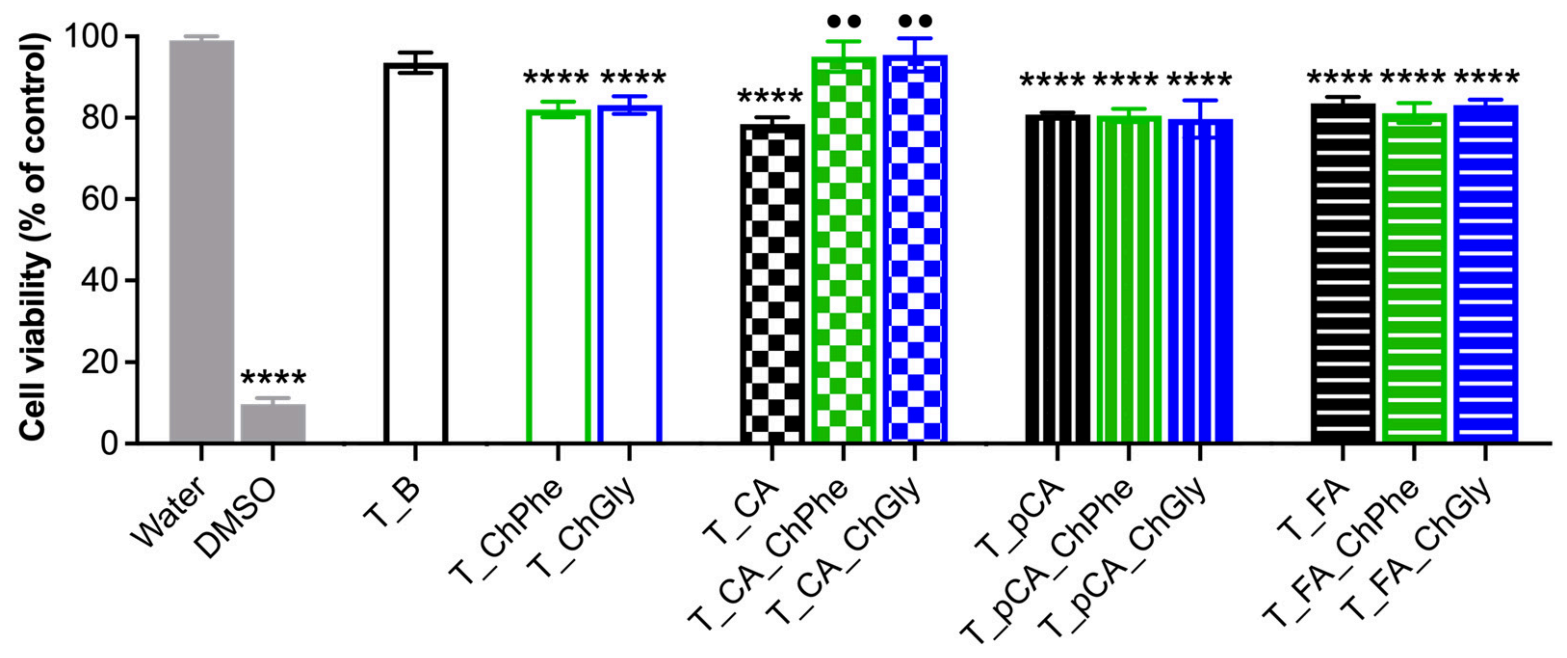
Cell viability of HaCaT cells after 24 h exposure to the developed transfersomes, as determined by the MTT assay (n = 2, mean ± SD, expressed as percentages of the non-treated control cells, **** *p* < 0.0001 compared with non-treated cells and •• *p* < 0.01 compared with T_CA formulation). Formulation designations combine the abbreviation for transfersomes (T) with, where applicable, the corresponding HCA (CA, Caffeic acid; pCA, *p*-Coumaric acid; FA, Ferulic acid) and/or IL (ChPhe for [Cho][Phe] and ChGly for [Cho][Gly]) (e.g., T_CA_ChPhe). T_B denotes blank transfersomes, while water and DMSO were used as controls. All transfersomes were diluted 20-fold, resulting in final concentrations of lecithin (2 mg/mL), Tween® 80 (0.105 mg/mL), CA (0.02 mg/mL without ILs and 0.07 mg/mL with ILs), pCA (0.035 mg/mL without ILs, 0.07 mg/mL with [Cho][Phe], and 0.075 mg/mL with [Cho][Gly]), and FA (0.03 mg/mL both with and without ILs).

The impact of transfersomes with and without HCAs and ILs on cell viability was probed. Since a topical skin application is envisioned for these nanosystems, a 2D culture of HaCaT cells was used as a model to evaluate cytotoxicity. None of the transfersome or transfersomIL formulations reduced cell viability below the 70% threshold for in vitro cytotoxicity, as defined by ISO 10993-5:2009 [50], under the tested conditions (Figure 6). Interestingly, CA-loaded transfersomILs had a lower impact on cell viability than the corresponding CA-loaded transfersomes, suggesting that CA exerts a protective effect at higher concentrations (0.07 vs. 0.02 mg/mL) when delivered in transfersomILs.

## 4. Discussion

The present study evaluated the impact of incorporating cholinium-based ILs, namely [Cho][Phe] and [Cho][Gly], into transfersomal systems loaded with HCAs with distinct biopharmaceutical properties. Overall, the results demonstrate that IL incorporation significantly influenced the performance of the developed nanocarriers, although the extent of these effects depended on both the IL and the HCA under study.

One of the most consistent effects of IL incorporation was the significant reduction in vesicle size. While conventional transfersomes exhibited mean sizes ranging from 118 to 136 nm, all transfersomILs presented significantly smaller sizes, between 69 and 79 nm, in line with our previous data [21]. This suggests that IL incorporation may promote the increase in vesicle curvature, possibly through modifications in lipid packing and bilayer organization, as previously described for both ILs under study [51,52]. Additionally, IL incorporation resulted in significantly more negative ZP values, indicating improved electrostatic stabilization, which is aligned with the reported accumulation of anions in the interfacial region of the vesicles [51,52].

The incorporation of ILs also improved the encapsulation performance for all HCAs, as reflected by increased EA and LC, as previously reported for rutin [21]. However, distinct effects were observed depending on the IL used. [Cho][Gly] consistently promoted higher EA, whereas [Cho][Phe] generally resulted in higher LC. These differences likely reflect the distinct physicochemical properties of the ILs and their interactions with both the HCAs and the vesicular membrane. While [Cho][Gly] may enhance HCA solubilization mainly in the aqueous phase, [Cho][Phe] may establish stronger interactions with the aromatic HCA structures and the lipid bilayer, potentially allowing greater HCA accommodation within the vesicular interfacial region.

Storage stability studies showed that all formulations underwent progressive changes over 60 days, mainly reflected by an increase in particle size, suggesting some degree of aggregation during storage. Although formulations containing ILs maintained more negative ZP values, the improved electrostatic stabilization was insufficient to completely prevent size enlargement. This suggests that electrostatic repulsion alone may not ensure long-term physical stability and that additional destabilization mechanisms, such as membrane rearrangement or vesicle fusion, likely contributed to the observed changes. In fact, it has already been demonstrated that cholinium- and imidazolium-based ILs can promote lipid bilayer fusion [38,39], although higher concentrations may be necessary. Interestingly, the impact of IL incorporation on stability appeared to be HCA-dependent, with FA-loaded transfersomILs displaying more promising results, highlighting the importance of HCA–IL–membrane interactions in determining formulation behaviour over time. Overall, these findings indicate that further formulation optimization is required to improve long-term physical stability.

The release studies further confirmed that IL incorporation affected the performance of transfersomes in a compound-dependent manner. Distinct release profiles were observed for the three HCAs, reflecting differences in their physicochemical properties and interactions with the vesicular systems. Notably, [Cho][Phe] delayed CA release, had minimal influence on pCA release, and promoted FA release. These results suggest stronger interactions between CA and the phenylalaninate anion, which may enhance CA retention within the transfersomal system. In contrast, the glycinate anion appeared to interact more strongly with pCA, delaying its release, while promoting the release of FA. Moreover, kinetic modelling indicated that the HCA release behaviour from transfersomes was predominantly governed by concentration-dependent and diffusion-controlled processes [43], as evidenced by the high correlation coefficients obtained with the first-order and Higuchi models. The poor fit of the Hixson–Crowell model suggests that changes in vesicle surface area or geometry during dissolution were not the predominant factors governing drug release [53]. The incorporation of cholinium-based ILs modulated the release kinetics as a function of the encapsulated HCA. For CA, [Cho][Phe] reduced the release constants while preserving the first-order and Higuchi release behaviour, whereas [Cho][Gly] shifted the release profile towards zero-order kinetics, indicating a constant release rate over time [43]. In contrast, both ILs maintained the kinetic behaviour of pCA but decreased the release constants, particularly [Cho][Gly], suggesting a slower release process. Conversely, IL incorporation increased the release rate of FA, with the greatest effect observed for [Cho][Gly]. These differences likely arise from the combined interactions of the HCAs and ILs with the phospholipid bilayer. Molecular dynamics studies have shown that cholinium-based ILs accumulate at the membrane interface and alter bilayer organization, with [Cho][Phe] partially intercalating into the membrane and [Cho][Gly] interacting mainly with the polar headgroup region [51,52]. In addition, the interaction of HCAs with phospholipid membranes is structure-dependent, as differences in the hydroxyl and methoxy substitution pattern influence their membrane affinity, partitioning, and lipid packing [54,55,56]. Together, these factors likely account for the compound-dependent differences in release kinetics observed in the present study. Nevertheless, further mechanistic studies are required, as neither the influence of EAs nor the combined effects of HCAs and ILs on these interactions have been investigated.

Concerning permeation, the performance of transfersomILs was benchmarked against both aqueous solutions, a vehicle in which HCAs exhibit limited solubility, and conventional transfersomes. The three HCAs are relatively lipophilic compounds and were incorporated into the nanosystems at concentrations corresponding to their solubility limits in the respective vehicles. For CA and pCA, transfersomILs enabled substantially higher drug loading owing to the increased solubility provided by the IL-containing formulations. Since FA was investigated at a later stage of the work, transfersomIL formulations containing FA were prepared at the same concentration as the aqueous and transfersomal formulations, to enable the differentiation between the effects arising from increased drug concentration and those attributable to the incorporation of the IL itself.

The permeation studies demonstrated that transfersomILs generally enhanced the flux of HCAs across both silastic and human epidermal membranes when compared with transfersomes. However, these differences did not reach statistical significance for T_pCA_ChPhe in human epidermis. [Cho][Gly] generally produced greater increases in permeation than [Cho][Phe]. These results indicate that the incorporation of ILs into transfersomal systems can improve transmembrane transport beyond the effects achieved by conventional transfersomes.

For CA and pCA, which were evaluated under saturated conditions, the thermodynamic activity of the permeant is expected to be similar across vehicles, provided that the vehicle does not significantly alter the membrane. Under these conditions, the steady-state flux should remain largely independent of the vehicle, reflecting a constant chemical potential of the permeant [57]. Therefore, the higher fluxes observed for most transfersomIL formulations cannot be explained solely by the increased solubility of the compounds in the formulations. Instead, they suggest an increase in the diffusion coefficient within the membrane, the partition coefficient between the formulation and the membrane, or both.

Interestingly, similar permeation enhancement trends were observed in both silastic and epidermal membranes. Since silastic membranes are relatively inert and are not expected to undergo significant structural changes in the presence of ILs, the comparable behaviour observed in both membrane models suggests that enhanced membrane partitioning is a major contributor to the increased permeation. In contrast, the epidermis contains a highly organized lipid matrix that may be susceptible to IL-induced perturbations. Therefore, the similar results obtained with both membrane models point towards partition-related effects as the dominant mechanism. Nevertheless, changes in the lipid organization of the stratum corneum cannot be excluded in the epidermal studies.

An exception to the overall trend was observed for the T_CA_ChPhe system, which did not exhibit higher fluxes than the aqueous saturated solution in either silastic or epidermal membranes. This result indicates that the increase in CA solubility provided by [Cho][Phe] was not translated into enhanced membrane transport. A plausible explanation is the existence of stronger interactions between CA and the phenylalaninate anion, including hydrogen-bonding and aromatic interactions, which may stabilize CA within the formulation, in line with the data obtained in release studies. As permeation is governed by thermodynamic activity rather than total drug concentration, these interactions could reduce the fraction of CA available to partition into the membrane, offsetting any benefit arising from the increased solubility. The observation of identical behaviour in both silastic and epidermal membranes further supports the hypothesis that the lack of enhancement is primarily related to formulation–drug interactions rather than membrane-specific effects.

The higher epidermal permeation of pCA compared with CA and FA may be attributed to its more favourable physicochemical profile for partitioning and diffusion across the skin barrier. Among the three HCAs, pCA has a slightly lower molecular weight, which can facilitate molecular mobility within the epidermal layers. In addition, its higher log D suggests a greater affinity for the lipid-rich domains of the stratum corneum, promoting more efficient partitioning into the membrane. At the same time, its higher aqueous solubility at 25 °C may improve its availability in the donor phase, supporting a stronger concentration gradient for permeation. This combination of adequate lipophilicity, good aqueous solubility, and smaller molecular size likely contributes to the enhanced epidermal permeation observed for pCA relative to CA and FA.

The superior performance of [Cho][Gly]-containing formulations may be attributed to a more favourable balance between solubilization and drug release. While [Cho][Gly] increases HCA solubility, its interactions with the permeants appear to be sufficiently weak to maintain a high thermodynamic activity and facilitate membrane partitioning. This interpretation is consistent with the permeation enhancement observed across all HCAs and in both membrane models, although the enhancement achieved with CA-encapsulated transfersomILs was modest.

Interestingly, conventional transfersomes did not improve permeation and, for CA and FA, yielded lower fluxes than the corresponding aqueous saturated solutions. This finding indicates that encapsulation within transfersomal vesicles alone does not necessarily enhance membrane transport. On the contrary, incorporation of the compounds into the vesicular structures may reduce the concentration of freely available permeant in the donor phase, thereby limiting partitioning into the membrane. Thus, the higher fluxes generally achieved with transfersomILs highlight the key role of ILs in overcoming this limitation and promoting permeation.

Finally, the close agreement between the permeation trends obtained with silastic and human epidermal membranes further supports the suitability of silastic as a predictive screening model for assessing HCA permeation and formulation performance. Despite the structural and compositional differences between the two membranes, the same ranking of formulations and permeation enhancement profiles was observed, indicating that silastic membranes can provide valuable mechanistic information during the early stages of formulation development.

The occlusive properties of transfersomILs were also assessed using an in vitro methodology. This assay measured water loss across silastic artificial membranes. When transfersomes were applied to these membranes, water loss decreased significantly compared with membranes treated with water. Interestingly, the occlusive effect was more pronounced in transfersomILs and was further enhanced in loaded transfersomILs. Incorporation of HCA into the lipid-based systems may lead to closer organization of the phospholipid bilayers, forming a denser structure that more effectively reduces water loss. A similar result was obtained in a study conducted with cerosomes [40]. These findings provided an indication that these systems can create a surface film on the membrane, which may support the added value of transfersomILs in future skin applications, given the essential role of lipids in maintaining the stratum corneum lipid barrier.

ILs are increasingly recognised as promising ingredients for topical and transdermal formulations, even in the case of permeants with high molecular weight such as polysaccharides, nucleic acids, proteins, and peptides [13]. However, despite their considerable potential for clinical application, particularly as penetration enhancers, the use of ILs remains limited by unresolved safety concerns [58]. To address this issue, we previously reported that 0.2% (*v*/*v*) of [Cho][Phe] and [Cho][Gly] is cytocompatible with both keratinocytes and renal cells [18,21,59]. These findings provided the rationale for incorporating these ILs at this concentration into transfersomes. To the best of our knowledge, this is the first study to evaluate the effects of transfersomILs on HaCaT cell viability. All formulations exhibited a promising safety profile for cutaneous application, maintaining cell viability at approximately 80% or higher under the tested conditions. Moreover, the similar viability observed for most loaded and unloaded transfersomILs suggests that the carrier system, rather than the encapsulated HCA, primarily determined the cellular response. An exception was observed for CA-loaded transfersomILs, which maintained cell viability close to 100% despite containing a higher CA concentration than the corresponding transfersomes. This effect may be associated with the antioxidant properties of CA [34], although the underlying mechanism remains unclear. Nevertheless, further studies are needed to confirm the biocompatibility of these nanosystems. These should include more advanced in vitro models, such as 3D skin models, and the assessment of additional safety endpoints beyond metabolic activity.

## 5. Conclusions

Transfersomes are both elastic and malleable vesicular systems, which are known to promote solubility, especially when paired with ILs, allowing for the creation of innovative formulations with promising features for topical application. Looking at the work developed and explained throughout this report, ILs proved to be particularly advantageous by providing nanosystems with suitable physicochemical properties compatible with skin applications, as well as enhanced permeation and occlusive properties. TransfersomILs showed promising cytocompatibility, but long-term stability was not fully achieved.

Overall, transfersomILs show promise as effective tools for enhancing the drug loading and solubility of polyphenols, such as the HCAs investigated in this work. Although significant progress has been made, further research on these nanovesicular systems is warranted. Future work should expand on the assays performed here and extend the investigation to other active pharmaceutical ingredients and bioactives, while also addressing key parameters such as stability, efficacy, and safety of use using advanced in vitro models and in vivo studies.

## Figures and Tables

**Table 1:** Quantitative composition of the produced transfersomILs and their corresponding controls in terms of HCAs and ILs. T_B corresponds to blank transfersomes; T_CA, to caffeic acid (CA)-loaded transfersomes; T_pCA, to *p*-coumaric acid (pCA)-loaded transfersomes; T_FA, to ferulic acid (FA)-loaded transfersomes; T_ChPhe, to transfersomes containing [Cho][Phe], and T_ChGly, to transfersomes containing [Cho][Gly]. Transfersomes containing an HCA and an IL are identified by combining the corresponding abbreviations (e.g., T_CA_ChPhe).

Formulation	Caffeic Acid ^1^	*p*-Coumaric Acid ^1^	Ferulic Acid ^2^	[Cho][Phe] ^3^	[Cho][Gly] ^3^
	Concentration (mg/mL)			% (*v*/*v*)	
T_B	-	-	-	-	-
T_ChPhe	-	-	-	0.2	-
T_ChGly	-	-	-	-	0.2
T_CA	0.4	-	-	-	-
T_CA_ChPhe	1.4	-	-	0.2	-
T_CA_ChGly	1.4	-	-	-	0.2
T_pCA	-	0.7	-	-	-
T_pCA_ChPhe	-	1.4	-	0.2	-
T_pCA_ChGly	-	1.5	-	-	0.2
T_FA	-	-	0.6	-	-
T_FA_ChPhe	-	-	0.6	0.2	-
T_FA_ChGly	-	-	0.6	-	0.2

^1^ Maximum solubility at 25 °C in water or water:IL mixture [18]. ^2^ Maximum solubility at 25 °C in water [18]. ^3^ Concentration at which cell viability remains above 80% in human keratinocytes [18].

**Table 2:** Physicochemical properties of the produced transfersomes in the absence and presence of an HCA and/or IL ([Cho][Gly] or [Cho][Phe]). (n = 3, mean ± SD; * *p* < 0.05, ** *p* < 0.01, and *** *p* < 0.001 compared with transfersomes without HCA and without IL (T_B)).

Formulation	Size (nm)	PDI	ZP (mV)	AE (%)	LC (%)
T_B	121 ± 4	0.21 ± 0.01	−25 ± 2	-	-
T_ChPhe	75 ± 2 ***	0.21 ± 0.01	−35 ± 2 *	-	-
T_ChGly	73 ± 4 ***	0.24 ± 0.02	−31 ± 2 *	-	-
T_CA	118 ± 2	0.22 ± 0.01	−20 ± 3	67.7 ± 1.3	0.45 ± 0.08
T_CA_ChPhe	73 ± 2 ***	0.21 ± 0.01	−44 ± 3 **	79.2 ± 0.8 *	1.88 ± 0.61 *
T_CA_ChGly	71 ± 3 ***	0.20 ± 0.01	−38 ± 3 *	87.5 ± 1.5 **	1.45 ± 0.08 *
T_pCA	123 ± 5	0.20 ± 0.01	−22 ± 3	70.5 ± 1.2	0.50 ± 0.17
T_pCA_ChPhe	79 ± 2 ***	0.25 ± 0.01	−48 ± 2 **	76.7 ± 0.6 *	2.10 ± 0.71 *
T_pCA_ChGly	73 ± 3 ***	0.23 ± 0.01	−39 ± 4 *	85.4 ± 0.2 **	1.97 ± 0.20 *
T_FA	136 ± 5 *	0.24 ± 0.03	−32 ± 2 *	71.2 ± 0.3	0.71 ± 0.02
T_FA_ChPhe	72 ± 3 ***	0.21 ± 0.07	−40 ± 3 **	75.1 ± 0.5 *	1.49 ± 0.07 *
T_FA_ChGly	69 ± 4 ***	0.23 ± 0.07	−42 ± 3 **	78.1 ± 0.8 **	1.29 ± 0.10 *

T_B, blank transfersomes; T_CA, caffeic acid (CA)-loaded transfersomes; T_pCA, *p*-coumaric acid (pCA)-loaded transfersomes; T_FA, ferulic acid (FA)-loaded transfersomes; T_ChPhe, transfersomes containing [Cho][Phe]; and T_ChGly, transfersomes containing [Cho][Gly]. Transfersomes containing an HCA and an IL are identified by combining the corresponding abbreviations (e.g., T_CA_ChPhe).

## Data Availability

The original contributions presented in this study are included in the article. Further inquiries can be directed to the corresponding authors.

## Supplementary Materials

The following supporting information can be downloaded at: https://www.mdpi.com/article/10.3390/pharmaceutics18080962/s1, Figure S1: Phase-contrast images of blank transfersomes (T_B), transfersomes containing [Cho][Phe] (T_ChPhe) and transfersomes containing [Cho][Gly] (T_ChGly). Table S1: Coefficient of determination (R2), rate constant (k), and release exponent (n) calculated by fitting the release profiles of loaded transfersomes, in the presence and absence of ionic liquids, with zero-order, first-order, Higuchi, Korsmeyer-Peppas and Hixson-Crowell kinetic models.

## Abbreviations

The following abbreviations are used in this manuscript:

% (*v*/*v*)	Percentage volume/volume
[Cho][AA]	Choline-amino acid
[Cho][Gly]	(2-hydroxyethyl)trimethylammonium glycinate
[Cho][Phe]	(2-hydroxyethyl)trimethylammonium phenylalaninate
AE	Association efficiency
CA	Caffeic acid
DMEM	Dulbecco’s modified Eagle’s medium
DMSO	Dimethyl sulfoxide
EA	Edge activator
FA	Ferulic acid
HaCaT	Human keratinocyte cell line
HCA	Hydroxycinnamic acids
ILs	Ionic Liquids
LC	Loading capacity
MTT	Thiazolyl blue tetrazolium bromide
PBS	Phosphate- buffered saline
pCA	*p*-Coumaric acid
PDI	Polydispersity index
PDMS	Polydimethylsiloxane
SD	Standard deviation
WL	Water loss
ZP	Zeta potential
