# Supplementary material for: TransfersomILs: A Synergy to Boost the Skin Delivery of Hydroxycinnamic Acids
Source: Pharmaceutics. 2026 Aug 5;18(8):962. doi: 10.3390/pharmaceutics18080962 (PMC13516245; doi:10.3390/pharmaceutics18080962)
Supplement: Supplementary file 1 [file pharmaceutics-18-00962-s001.zip › pharmaceutics-4439904-supplementary.pdf]

Supplementary Data

TransfersomILs: a synergy to boost the skin delivery of hydroxycinnamic acids

Ana Júlio [1], Marta B. Martins [1], Teresa Martinho [2], João Vieira [1,3], Nuno Saraiva [1], Catarina Rosado [1,*] and Catarina Pereira-Leite [1,4,*]

[1] CBIOS, ECTS, Universidade Lusófona, Campo Grande, 376, 1749-024 Lisboa, Portugal; ana.julio@ulusofona.pt (A.J.), p8490@ulusofona.pt (M.B.M.), p6769@ulusofona.pt (J.V.), nuno.saraiva@ulusofona.pt (N.S.).
[2] ECTS, Universidade Lusófona, Campo Grande, 376, 1749-024 Lisboa, Portugal.
[3] Universidad de Alcalá, Departamento de Ciências Biomédicas, Ctra. Madrid-Barcelona km. 33,600, 28805 Alcalá de Henares, Madrid, España.
[4] Laboratório Associado para a Química Verde (LAQV), REQUIMTE (Rede de Química e Tecnologia), Faculty of Pharmacy, University of Porto, Rua de Jorge Viterbo Ferreira n. 228 4050-313 Porto, Portugal.
* Correspondence: catarina.rosado@ulusofona.pt (C.R.) & catarina.leite@ulusofona.pt (C.P.-L.)

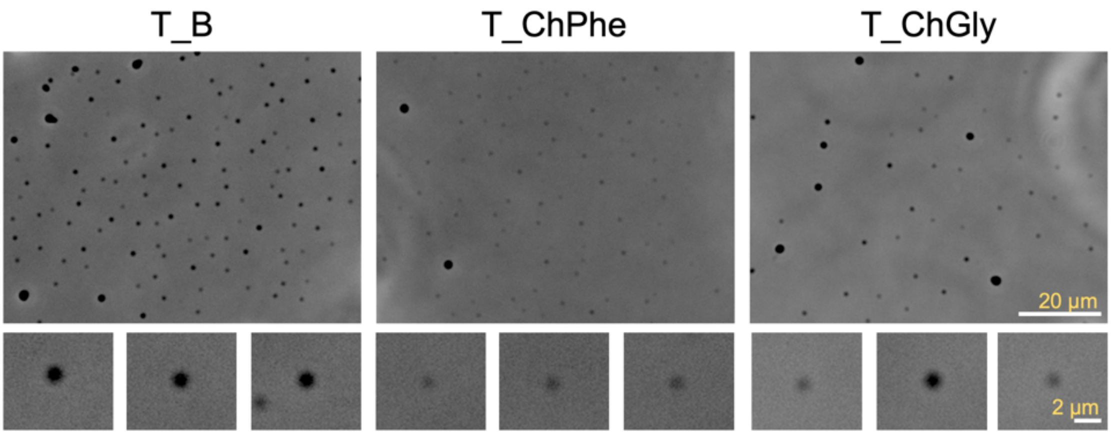

Figure S1. Phase-contrast images of blank transfersomes (T_B), transfersomes containing [Cho][Phe] (T_ChPhe) and transfersomes containing [Cho][Gly] (T_ChGly). Image acquisition was performed with an Axio Observer microscope (Zeiss, White Plains, NY, USA) with an x40 objective using ZEN blue software (Zeiss). Scale bar 20 μm (upper panel) and 2 μm (lower panel).

Table S1. Coefficient of determination (R^2), rate constant (k), and release exponent (n) calculated by fitting the release profiles of loaded transfersomes, in the presence and absence of ionic liquids, with zero order, first order, Higuchi, Korsmeyer-Peppas and Hixson-Crowell kinetic models.

Formulation	Zero order		First order		Higuchi		Korsmeyer-Peppas		Hixson-Crowell	
	R^2	k	R^2	k	R^2	k	R^2	k (n)	R^2	k
**T_CA**	0.9425	6.41	**0.9824**	**-0.15**	0.9711	26.63	0.9554	9.28 (1.01)	0.7191	-0.24
**T_CA_ChPhe**	0.9397	3.94	0.9760	-0.06	**0.9778**	**16.46**	0.9239	6.21 (1.01)	0.6943	-0.20
**T_CA_ChGly**	**0.9853**	**5.82**	0.9172	-0.14	0.9637	23.56	0.9563	10.91 (0.79)	0.7281	-0.22
**T_pCA**	0.9207	7.10	0.9646	-0.28	**0.9748**	**29.90**	0.9403	10.30 (0.94)	0.6890	-0.24
**T_pCA_ChPhe**	0.8844	6.74	**0.9839**	**-0.26**	0.9763	28.96	0.9707	17.43 (0.81)	0.6237	-0.22
**T_pCA_ChGly**	0.9530	4.96	**0.9914**	**-0.09**	0.9908	20.68	0.9881	14.94 (0.62)	0.6456	-0.20
**T_FA**	0.9717	4.62	**0.9849**	**-0.08**	0.9643	18.84	0.9823	8.25 (0.80)	0.738	-0.21
**T_FA_ChPhe**	0.9533	5.41	**0.9933**	**-0.11**	0.9816	22.47	0.9277	9.99 (0.89)	0.6929	-0.22
**T_FA_ChGly**	0.9692	6.75	**0.9777**	**-0.17**	0.9568	27.43	0.9724	6.66 (1.13)	0.7796	-0.25

T_CA, caffeic acid (CA)-loaded transfersomes; T_pCA, p-coumaric acid (pCA)-loaded transfersomes; T_FA, ferulic acid (FA)-loaded transfersomes; T_ChPhe, transfersomes containing [Cho][Phe]; and T_ChGly, transfersomes containing [Cho][Gly]. Transfersomes containing an HCA and an IL are identified by combining the corresponding abbreviations (e.g., T_CA_ChPhe).
